# Knowledge-based biomedical Data Science

**DOI:** 10.3233/DS-170001

**Published:** 2017-12-08

**Authors:** Lawrence E. Hunter

**Affiliations:** Computational Bioscience, University of Colorado School of Medicine, Aurora, CO 80045, USA Larry.Hunter@UCDenver.edu; ORCID: https://orcid.org/0000-0003-1455-3370

**Keywords:** Ontology, knowledge representation, reasoning, inference, machine learning, text mining, explanation

## Abstract

Computational manipulation of knowledge is an important, and often under-appreciated, aspect of biomedical Data Science. The first Data Science initiative from the US National Institutes of Health was entitled “Big Data to Knowledge (BD2K).” The main emphasis of the more than $200M allocated to that program has been on “Big Data;” the “Knowledge” component has largely been the implicit assumption that the work will lead to new biomedical knowledge. However, there is long-standing and highly productive work in *computational* knowledge representation and reasoning, and computational processing of knowledge has a role in the world of Data Science.

Knowledge-based biomedical Data Science involves the design and implementation of computer systems that *act as if they knew* about biomedicine. There are many ways in which a computational approach might act as if it knew something: for example, it might be able to answer a natural language question about a biomedical topic, or pass an exam; it might be able to use existing biomedical knowledge to rank or evaluate hypotheses; it might explain or interpret data in light of prior knowledge, either in a Bayesian or other sort of framework. These are all examples of automated reasoning that act on computational representations of knowledge. After a brief survey of existing approaches to knowledge-based data science, this position paper argues that such research is ripe for expansion, and expanded application.

## 1. Representations of biomedical knowledge

All computational approaches to knowledge require specification of how the computer system *represents* knowledge internally, and how it might compute with those representations to produce outputs (often called, perhaps metaphorically, *reasoning*). Classic descriptions of knowledge representation and reasoning systems, e.g. [16] focus on what ontological commitments a knowledge representation makes, what inferences are possible with it, and, sometimes, which of those inferences can be made efficiently. These issues remain useful in thinking about how knowledge representation and reasoning play a role in today’s data science environment.

As [16] pointed out, knowledge representations entail ontological commitments. Adoption of existing ontologies, rather than creating idiosyncratic or single-use ontologies provides significant advantages for reproducibility in scientific research, for inter-operability, and in avoiding pitfalls in the modeling of knowledge. A great deal of work has been done in biomedical ontology (e.g. [2,36,39,41,45] and many others), and these increasingly mature ontological resources form an important basis for knowledgebased data science. Community-curated ontologies (such as those meeting the Open Biomedical Ontologies (OBO) Foundry criteria [42]) capture a consensus view of the entities and processes involved in biology, medicine and biomedical research, analogous to how nomenclature committees systematize naming conventions.While not meeting all of the criteria of the OBO Foundry, terminological resources such as UMLS [30], Snomed-CT [7] and the NCI thesaurus [19] have also been used to provide useful pseudo-ontological foundations for knowledge representations.

While ontologies identify the basic elements from which a knowledge representation is constructed, they are agnostic about the mechanisms by which ontological units are assembled into representations of knowledge. Building on decades of work in artificial intelligence research, the W3C produced a collection of international standards for assembling ontological entities into assertions and managing collections of assertions, together referred to as the Semantic Web. The focus of the Semantic Web standards is to make it possible to link web elements with shared meaning, and is sometimes described as the *Linked Data* paradigm. The Semantic Web builds on the standard Resource Description Framework (RDF), which provides a way to link three uniform resource identifiers (URIs) to specify a pair of entities and a relationship between them (forming an RDF “triple”). Collections of triples form a graph, and a computational mechanism for managing such collections is called a triple store. The Semantic Web standards also define RDF Schemas (RDFS) and a Web Ontology Language (OWL) which facilitate richer knowledge representations, SPARQL, which provides a query language for interrogating RDF graphs or triple stores, and the Simple Knowledge Organization System (SKOS), which provides a basic ontology, including simple semantic relationships. While the Semantic Web standards are intended to be general representation tools for all knowledge (e.g. RDF for facilitating exchange of research data), the combination of Semantic Web standards and biomedical ontologies are the basis of most current biomedical knowledge representation systems.

## 2. Knowledge-based inference

Representations of knowledge are sterile without use. Although human visualization of computationally represented knowledge (e.g. [32]) can be useful, the primary use of computationally represented knowledge is inference. There are many forms of inference, and thousands of publications describing computational methods of reasoning. Although too broad to survey here, a brief introduction to the types of knowledge-based inference common in biomedical applications gives some idea of its potential.

### 2.1. Logical inference

Computational logical inference is a mapping from a base set of assertions to create additional assertions that are entailed by the base. While deductive reasoning is the classic form of logical inference, it is, in general, computationally intractable. Various restricted forms of deductive inference, such as those based on description logics, have better computational performance, at the cost of greatly restricting the utility of the inferences. Description logics, for example, are limited to inferring subsumption relationships based on necessary and sufficient class definitions. Contemporary applications of description logic inference in biomedical knowledge representation have been successful primarily in checking for modeling errors (e.g. [8,26]), although some other applications have been attempted (e.g. [9,22,23]).

Deductive retrieval is a special case of deductive inference, where the inference is to compute whether a set of logical axioms and base assertions can be combined to satisfy a query; the programming language Prolog and the W3C standard for the Semantic Web Rule Language (SWRL) are examples of approaches to deductive retrieval. Triple stores extended with deductive retrieval are much more valuable than those that can retrieve only queries that match exactly. Several knowledge-bases of biomedicine based on these technologies have been developed (e.g. [3,6,31,48]), and their uses extend beyond deductive retrieval alone.

### 2.2. Inference from ontology annotation

In addition to the creation of biomedical ontologies, a great deal of effort has gone into annotating genes and other biological entities to ontological categories. Gene Ontology annotations of genes and gene products figure prominently in major databases such as UniProt and the Mouse Genome Informatics. These annotations provide a quick summary of knowledge about gene function, subcellular localization and biological processes. By far the most common application of computational representations of knowledge to problems in biomedicine is enrichment analysis, see e.g. [24,43,46]. Enrichment analysis generates hypotheses about the concerted functions of collections of genes by testing for annotations that occur more frequently in the collection than would be expected by chance. Ontology annotation directly supports other sorts of knowledge-based inference as well. For example, phenotype annotations play a major role in mapping between human disease and animal models (e.g. [28,34,35]). Formal representations of metabolic pathways (e.g. [18,27]) have been used to analyze metabolomic data and support metabolic engineering.

### 2.3. Inference from the biomedical literature

Despite the rapid growth of databases with ontological annotation, the main and by far the largest repository of biomedical knowledge remains the published literature. An important domain of knowledge-based data science involves natural language processing with the goal of producing computational representations of the knowledge in the literature. The most basic of these approaches involves tagging passages in the literature with ontological terms (e.g. EuroPMC’s SciLite annotations, or [20]). Computational methods to identify semantically well-defined entities in the literature support further analysis that identifies links both among different documents in the literature (e.g. [52]) and between entities in the literature and database entries about them (e.g. [37]). More ambitious literature mining goals involve producing more complex knowledge representations directly by processing natural language documents, e.g. [15,49], although significant improvements in performance are likely to be necessary before the results of such processing find widespread use in biomedical research. Text mining approaches applied to clinical records and social media, e.g. for pharmacovigilance applications, have also made significant strides recently [17]. The best performing text mining systems themselves often use representations of prior knowledge to drive understanding of text.

Natural language processing systems have also been used to support automated question answering. Perhaps the most well known of these efforts is IBM’s Watson system [12], which has found significant biomedical application. Many other computational systems for question answering, targeted to biomedical researchers and clinicians, have been fielded, e.g. as reviewed in [1,4]. Computational approaches to building systems that can answer biomedical exam questions have also been developed, e.g. [14].

### 2.4. Hypothesis generation, evaluation and modification

Perhaps the oldest method of computing with knowledge is Bayesian inference [21]. By providing a quantitative framework for the idea that observations consistent with prior knowledge are more likely than ones that contradict it, Bayesian reasoning has provided a basis for knowledge-based computation long before computation was automated. Contemporary computers provide the power necessary to support more elaborate Bayesian inference, including model selection as well as estimating model parameters [13].

Network-based inference, such as link prediction or community finding, have been successfully applied to generate significant biomedical hypotheses. Systems that compute over representations of knowledge of biomedicine have been used to propose as yet unobserved relationships among biological entities, e.g. for drugs [33], microRNAs [51], diseases [44] and proteins [47]; some of these predictions have been empirically validated, e.g. [25].

Perhaps the most exciting potential for knowledge-based computational systems is in the development and refinement of mechanistic explanations of biomedical phenomena. The vast scope and rapid evolution of the biomedical literature, combined with the breakdown of disciplinary boundaries driven by genome-scale research has made it increasingly difficult for researchers to effectively assimilate all the knowledge potentially relevant to interpreting the results of their own experiments. Although most computational approaches aim to provide material for the *Results* section of a paper, a few are beginning to target the *Discussion* section as well. While no knowledge-based computer system has repeatedly generated important biomedical hypotheses *de novo*, promising proof-of-concept systems include systems to generate hypotheses from the literature [40] and those aimed at hypothesis generation or refinement from data [11,38], as well as mixed initiative human-computer hypothesis generation [29]. Although it remains aspirational, the synthesis of computational simulation with knowledge-based generation and refinement of hypotheses has received substantial interest from funding agencies [50].

## 3. Open challenges in knowledge-based Data Science

As is clear from the NIH BD2K experience, computation over knowledge is a less widespread research focus than analysis of big data, and to date has had less impact in biomedicine. Certain applications, such as enrichment analysis and link prediction, have found widespread use in biomedical research. Text mining systems are increasingly deployed in areas such as helping clinicians keep up with rapidly changing clinical data [10] and pharmacovigilance. However, there are significant challenges to realizing the potential for knowledge-based data science. Perhaps the foremost among these is the knowledge acquisition bottleneck: human curation, even for the relatively simple task of annotation of genes to gene ontology terms is difficult to scale [5]. Alternatives to manual curation, including applications of text mining and machine learning, have shown promise, but are still far short of human-like performance. Another important understudied question is how to represent what is *not* known: any scientist can describe gaps, ambiguities and uncertainties in existing knowledge, yet there are few computational methods capable of representing, let alone reasoning about, such ignorance.

Even more challenging than developing representations of what is already known is the application of that knowledge to the pressing problems of biomedical research. Existing inference methods are far short of the range and creativity of human experts in developing potential explanations, generating significant hypotheses, and generally interpreting results in light of previous knowledge. Many promising inference methods scale poorly, and are constrained in their ability to harness large knowledge-bases by the extremely large computational loads involved. Even deductive retrieval systems can be computationally intractable over large knowledge-bases; more complex forms of inference hit the limits of current hardware with even smaller knowledge-bases. The Semantic Web standard was developed largely with description logic inference in mind; while it provides a solid foundation for knowledge representation systems, representational transformations may improve the efficiency of other sorts of inference.

Perhaps the biggest challenges in knowledge-based data science are in developing the vision for what such a system could effectively contribute to biomedical research. Is it possible to build computational systems that bring to bear disparate yet relevant facts from across all biomedical disciplines and scales, exploiting their ability to process far more information than any individual human being? Could such a system make sound judgements ranking alternative hypotheses based on an exhaustive comprehension of the literature? Is it possible for computational systems to generate significant and novel mechanistic and pathomechanistic hypotheses about open questions in biomedicine? It is positive answers to questions like these that will drive knowledge-based data science into the mainstream of biomedical research.
